# Evaluation of pH and residual gastric volume after colon preparation with mannitol: prospective randomized study comparing procedure performed after 3 hours *versus* 6 hours

**DOI:** 10.6061/clinics/2020/e1847

**Published:** 2020-11-02

**Authors:** Renzo Feitosa Ruiz, Jose Jukemura, Paulo Roberto Arruda Alves, Marcos Eduardo Lera dos Santos

**Affiliations:** I Hospital Alemao Oswaldo Cruz, Sao Paulo, SP, BR; II Departamento de Gastroenterologia, Faculdade de Medicina (FMUSP), Universidade de Sao Paulo, SP, BR

**Keywords:** Mannitol, Colonoscopy, Bowel Preparation, Gastric fluid, Fasting Period

## Abstract

**OBJECTIVES:**

Our goal was to compare the hydrogen potential (pH) and residual gastric volume (RGV) of patients undergoing colonoscopy after 3 and 6 hours of colon preparation with mannitol.

**METHODS:**

We described a prospective randomized trial with a 50:50 allocation rate of two distinct times of colonoscopy after colon preparation with 10% mannitol. We included outpatients aged over 18 years, with no history of gastric surgeries and an American Society of Anesthesiologists (ASA)-rated anesthetic risk below III. Colonoscopy was performed after upper digestive endoscopy at two different times: 3 versus 6-hour after mannitol ingestion. During upper gastrointestinal endoscopy, we measured RGV and evaluated pH with a digital pH meter. Clinical trials.gov: 71123317.9.3001.0065

**RESULTS:**

We randomized a total of 100 participants to the 3 and 6-hour groups, with the patients in the 6-hour group being younger and presenting a higher body mass index (BMI). The intervention did not result in any statistically significant differences between the two groups, neither for the RGV (*p*=0.98) or the pH (*p*=0.732). However, the subgroup of patients with diabetes mellitus showed statistically significant higher RGV values in the 3-hour group.

**CONCLUSION:**

There was no difference between RGV and pH values at 3 *versus* 6-hour after bowel preparation with mannitol, except for RGV in diabetic patients at 3 hours. As prolonged fasting protocols may result in adverse events such as dehydration and electrolyte imbalance, we can infer that colonic preparation with mannitol in shorter fasting periods, such as 3 hours, can be adopted safely and routinely.

## INTRODUCTION

Healthcare providers and the research community consider colonoscopy as a gold-standard for conditions requiring the mucosal imaging of the entire colon, playing an essential role in the diagnosis and treatment of colonic disorders. Adequate colon cleansing, also known as bowel preparation, is critical for the complete visualization of the colonic mucosa, increasing its diagnostic accuracy (1). Insufficient cleansing or preparation is associated with a low diagnostic yield, a higher rate of canceled procedures, corresponding increased costs, lengthy procedures, and a higher risk of complications. The existing literature has identified multiple factors contributing to suboptimal bowel preparation, including comorbidities, age, gender, use of antidepressants, education level, history of abdominal surgery, non-compliance with preparation instructions, and the timing of bowel preparation before the colonoscopy (2,3). Recent studies suggest that the time interval between the last intake of bowel preparation medication and the start of colonoscopy, is a significant factor in determining the efficacy of cleansing (4). For example, a short interval of 4 to 6 hours results in a better quality of bowel cleansing (4,5). The 2019 European Digestive Endoscopy Society Guidelines for Intestinal Preparation recommended to perform colonoscopies within 5 hours after the last dose of bowel preparation and to begin procedure at least 2 hours after completion of bowel preparation (6).

Along with appropriate timing of administration, bowel preparations need to be safe, palatable, and efficacious (2). Mannitol is a hyperosmolar liquid reported to be comparatively effective and well-tolerated as polyethylene glycol (PEG) and oral sodium phosphate (OSP) (7-10). Healthcare providers in Brazil and other parts of the world extensively administer mannitol due to its effective bowel cleansing, better tolerance, low complication rates, and low costs (8-11). Therefore, it is critical to compare outcomes among patients that receive mannitol before endoscopic procedures at different time points.

Without sufficient bowel preparation, endoscopic procedures are more challenging due to decreased visibility, which leads to more complications. One of the most concerning issues is the bronchial aspiration of gastric fluids during colonoscopy or upper digestive endoscopy procedures done with anesthesia services compared with standard sedation (0.14% *versus* 0.10%; *p=*0.02) (12). Nonetheless, it is possible to reduce the risk and consequences of gastric fluid aspiration by minimizing residual gastric volume (RGV), increasing gastric emptying, and increasing the gastric content’s hydrogen potential (pH) (13). Studies suggest that fasting time before anesthesia is the only significant factor in determining RGV. A study analyzed the gastric emptying after sodium phosphate bowel preparation by transabdominal ultrasound before colonoscopy. The result reported that 150 min of fasting before the procedure can lead to a RGV of 20 mL in 92% of the cases, however, this volume is not reduced further by an additional 10 hours of fasting (14). The literature demonstrates that in a PEG split-dose regime, final bowel preparation administered between two and 3 hours before colonoscopy resulted in similar RGVs, as when a full dose is taken a night before the procedure (2).

Nevertheless, several institutions still recommend and practice pre-anesthetic fasting of 6 to 8 hours to prevent an event of anesthetic aspiration, even knowing that the American Society of Anesthesiologists (ASA) guideline recommends only 2 hours fasting for clear liquids (15). According to a meta-analysis, clear liquids given 2 to 4 hours before the procedure result in a lower risk of aspiration, with gastric volumes below 25 mL and pH greater than 2.5 (15). Another study confirmed that drinking 330 mL of water approximately 2 hours before an endoscopy did not change the volume and pH of gastric content (13). Therefore, to reduce the risk associated with RGV and pH concerning aspiration, it is essential to optimize the pre-anesthetic fasting time before colonoscopy. Despite all of this literature, there is a lack of studies evaluating RGVs and pH in patients undergoing mannitol bowel preparation for colonoscopy after clinically meaningful periods.

Considering the previous literature and corresponding gaps, this study’s main goal was to conduct a randomized clinical trial comparing pH and RGVs between two different pre-colonoscopic regimens, namely a 3 *versus* 6-hour ingestion of mannitol before the colonoscopy. The hypothesis was that there would be no differences in the RGV and pH value between the two regimes.

## MATERIAL AND METHODS

We conducted a randomized clinical trial with a parallel assignment of subjects using a 50:50 assignment ratio. The study followed the CONSORT recommendations (16). The main objective was to compare pH measurement and the RGV after mannitol intake at two different intervals: 3-hour *versus* 6-hour period for bowel preparation. The study sample involved patients referred for upper digestive endoscopy and colonoscopy at the Department of Digestive Endoscopy (State Hospital of Sapopemba, Brazil). We did not conduct any modification to the trial design after initiating the study. Specifically, we followed the same inclusion criteria and protocols during active recruitment.

The Institutional Review Boards of the State Hospital Sapopemba and the Department of Gastroenterology of the University of São Paulo approved this study. All patients provided informed consent. We registered the trial under the Brazilian trial platform (71123317.9.3001.0065).

### Study subjects

We included ambulatory patients scheduled to undergo upper digestive endoscopy and colonoscopy on the same day, as requested by their doctors. Inclusion criteria involved patients of 18 years or older, with no previous gastric surgeries, having an Anesthesiologists (ASA) risk of less than III, and fasting from solid food for at least 8 hours. We excluded patients presenting with compromised walking, pregnant women, and those recently using anti-acids. We also excluded patients presenting with a history of atrophic gastritis or obstructive gastric lesions, recent use of anti-emetics, and not evacuating bowel after 6 hours of mannitol ingestion. We initiated participant accrual in May of 2017 and concluded the study in July of the same year.

### Study interventions

The primary study intervention was a bowel preparation with mannitol in two forms: 3 *versus* 6-hour before the endoscopic procedures. All remaining study procedures other than the time of ingestion were identical between both the randomized groups. These included the oral intake of 1,000 mL solution at 10% mannitol (500 mL of mannitol at 20% with 500 mL of water). The randomization assigned the mannitol solution to be drunk either 3 or 6 hours before the study evaluation. We offered the solution to patients when they were randomized to each group. We instructed all the patients to drink the solution until 3 or 6 hours before the procedure. Participants were allowed to ingest clear liquids until the last mannitol intake, after which they fasted until the colonoscopy. We defined fasting time as the interval from the completion of bowel preparation to colonoscopy.

For safety reasons and following clinical judgment, we administered intravenous hydration on a case-by-case basis if an individual showed signs of dehydration. In both groups, we advised participants to walk after ingesting the mannitol.

Two clinicians assisted by two nurse technicians performed all study procedures on all subjects. All personnel involved in the study had a certification in Advanced Cardiovascular Life Support procedures. Moreover, we did not implement blinding for clinicians, nurses, and patients. Blinding involved only the researcher responsible for data analysis.

Intravenous drugs for deep sedation included standard doses of 2.5 mg midazolam, 50 mcg fentanyl, and 0.5 mg/kg propofol. Participants were monitored throughout the procedure, using pulse oximetry and blood pressure measurement. We administered oxygen flow at a rate of 3 L/min through a nasal cannula. A 10-20 mg every 10 s of propofol was administered to all patients until we attained the desired level of sedation.

### Endpoints

The outcomes of interest were the values of pH and RGV.

**Figure 1:**
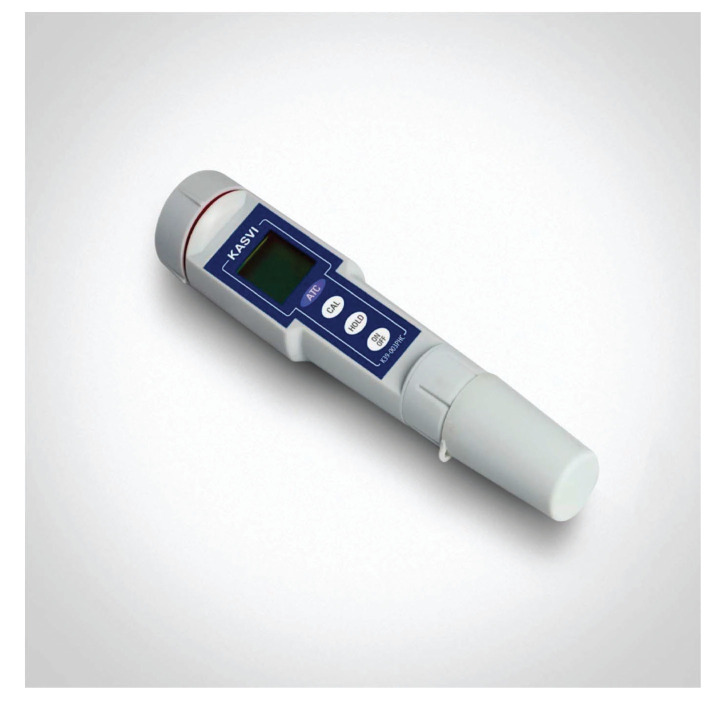
Portable digital meter.

After the aspiration of the residual gastric liquid by direct visualization using a gastroscope, we measured its volume through a calibrated secretion container (rigid PVC [polyvinyl chloride] bottle, calibrated every 10 mL, with a capacity of 1000 mL) in milliliters. We selected a safe limit for the RGV of 25 mL, as the literature reports that it is associated with a low risk of aspiration (15). The pH was measured using a digital pH meter (Accuracy: ±0.06; Resolution: 0.01; Measuring Range: 0.00-14.00; Dimensions: 158 x 40 x 34 mm; 9 V battery; Temperature Range: 5-35°C) (Figure 1).

### Sample size

Considering an alpha error of 5%, we estimated that a sample size of at least 50 patients per group was necessary to yield a 90% power to detect a difference in gastric pH of at least 0.5, assuming a standard difference of 0.30 (17).

### Statistical methods

We performed an exploratory analysis of all variables to evaluate their frequency, percentage, and near-zero variance for categorical variables, including sex, comorbidities, ASA category, extended hiatus, and Boston scale. We assessed the distribution for numeric variables, including pH, RGV, age, body mass index (BMI), and corresponding missing value patterns (18). Near-zero variance was evaluated as categorical variables that presented a small percentage of a given category. In response to these findings, we conducted variable transformations, dummy coding for variables with distributions that were not normal, variable re-categorization or removal for near-zero variation, and different application of imputation algorithms to address variables with missing values (19).

We performed randomization with a dice right after informed consent. We did not implement participant blinding as subjects were aware of when the preparation occurred. The primary investigator performed the analyses, with the dataset having false information regarding the treatment arms to maintain blinding.

We conducted the balance across arms at baseline, and comparisons between interventions using a series of inferential, unadjusted tests. Specifically, we evaluated differences in baseline variables between intervention arms using t-tests for the numeric variables (age, BMI, volume, pH, and the logarithm of volume). We then used chi-square tests for the categorical variables, including the Boston scale, ASA criteria, hernia, and enlarged hiatus. In case of imbalances, the protocol called for adjustments was through generalized linear models using a Gaussian distribution family (multiple linear regression) for numeric outcomes or a binomial distribution family (logistic regression) for dichotomous endpoints. These models evaluated the association between all previously reported outcome variables and the interventions, accounting for the baseline differences that remained unbalanced after randomization. We reported results as predicted means for numeric outcomes, along with 95% confidence intervals. We considered results statistically significant when confidence intervals did not overlap between different estimates. Finally, we performed subgroup analyses by testing the same association between the intervention and outcomes within specific subgroups of the sample. We split patients by BMI (above *versus* below or equal to 26.4 kg/m^2^, which represents the median BMI value for this sample) and the Boston scale score (20) (below *versus* equal to nine, which corresponds to the maximum possible score for this scale and represents a perfectly clean colon without any residual liquid). We also split patients by the ASA score (healthy *versus* those with a mild systemic disease based on patients-ASA risk classification as I and II respectively), presence of diabetes, and hyperthyroidism. Since these are post hoc analyses, we should interpret them with caution.

## RESULTS

We screened a total of 119 patients until the allocation in the groups was 50:50. Therefore 19 of them were excluded since they had taken anti-emetics before the endoscopic procedure, presented a history of atrophic gastritis or obstructive gastric lesion, or had no bowel evacuation after mannitol ingestion.

**Figure 2:**
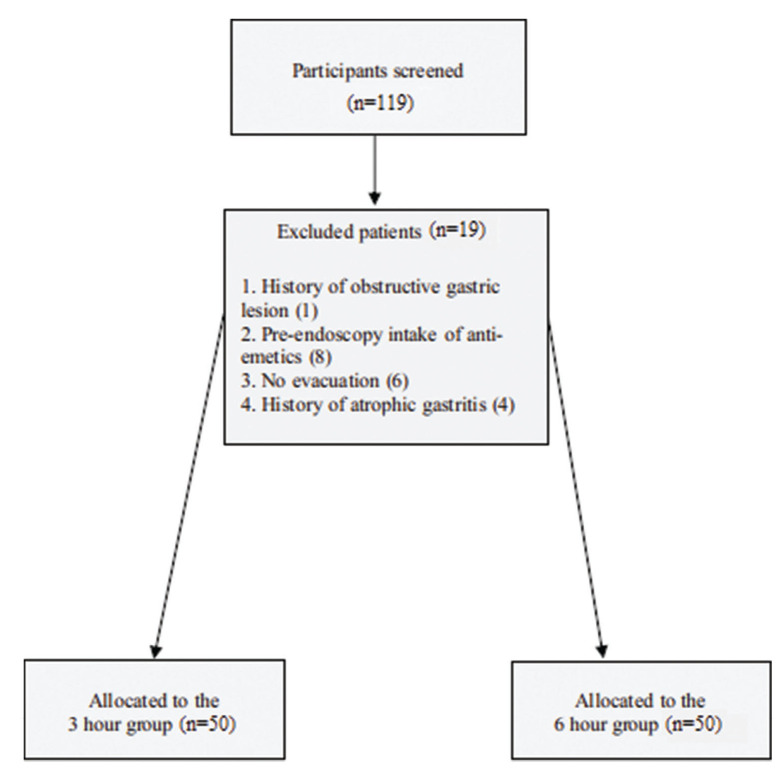
Study flowchart.

Figure 2 presents the sample's information, including the total number of subjects in the study, exclusions, and the total number of patients included in the analysis.

A total of 100 participants were randomized, 50 assigned to the 3-hour arm, 50 to the 6-hour arm. The mean age of the total sample was 55.7 years, 51% of them being females. When comparing the two interventions, those in the 6-hour group were younger (51.7 *versus* 59.7 years) and presented with higher BMI (28.5 *versus* 26.4 kg/m^2^) (Table 1). Our results indicated a balance between the randomized arms for all measured baseline variables, except for age and BMI. The exclusion criteria likely caused this imbalance. Consequently, we adjusted all subsequent analyses for these two variables to address any potential confounding in results.

**Figure 3:**
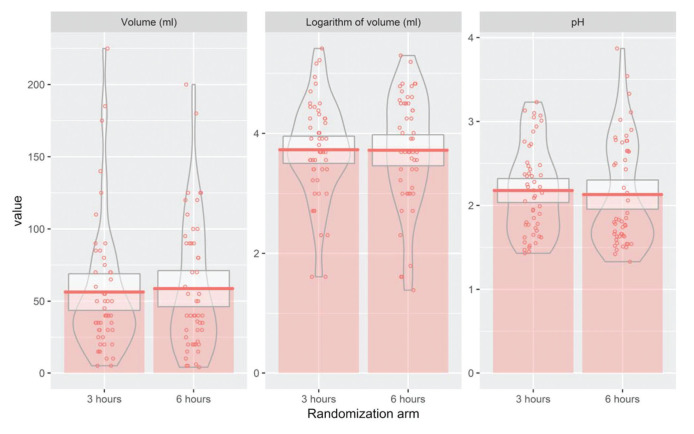
Residual gastric volume in 3 and 6-hour arms.

While evaluating the association between the two interventions and outcome measures through a multiple regression model, we found no significant differences between the 3-hour and 6-hour arms for any outcome measure, namely RGV and pH (Table 2). Since RGV presented a non-normal distribution, we also analyzed the logarithmic transformation of this endpoint. However, to improve clinical interpretability, we exponentiated the results of the logarithm of volume after applying the multiple linear regression model (Table 2). No statistically significant differences were found after the transformation, confirming the initial results (Figure 3). Next, we evaluated the predicted means for each outcome, including RGV (mL), the logarithm of RGV (mL), and pH for each of the eight subgroups. Our results indicated that diabetic patients in the 3-hour arm presented with significantly higher RGVs than those in the 6-hour arm. This association was confirmed when conducting the analysis using the log-transformed variable (Table 3).

**Figure 4:**
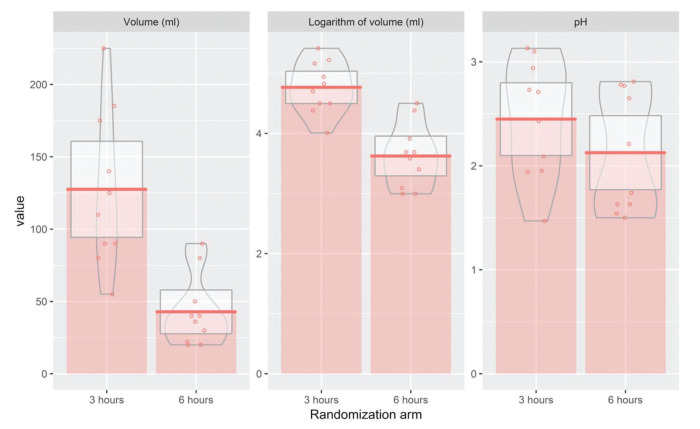
Association between the two interventions and residual gastric volume (mL), logarithm of residual gastric volume (mL), and pH for patients with diabetes mellitus.

When evaluating outcomes for the subgroup with diabetes mellitus comorbidity, the 3-hour arm presented a significantly higher volume (127 *versus* 43.4 mL comparing the 3 *versus* 6-hour groups). We observed a similar result when evaluating the logarithm of volume (116 *versus* 37.8). However, when we compared the pH between both interventions, no statistically significant differences were observed (Figure 4).

**Figure 5:**
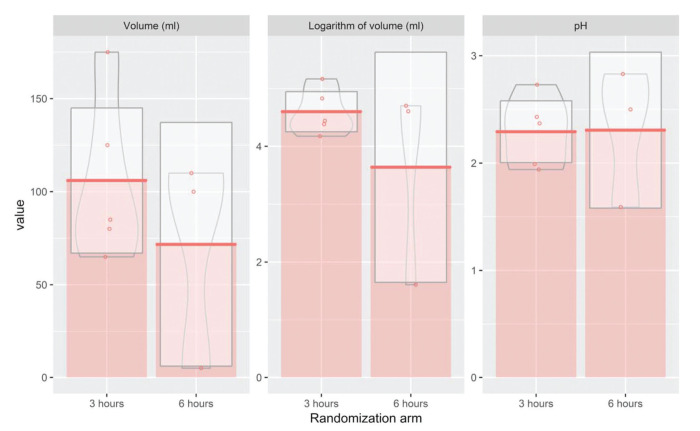
Association between the two interventions and residual gastric volume (mL), logarithm of residual gastric volume (mL), and pH for patients with hypothyroidism.

Regarding the subgroup analysis conducted among patients with hypothyroidism, the 3-hour arm presented a higher volume (mL) and logarithm of volume compared to the 6-hour arm (111 *versus* 63.2 and 139 *versus* 21.7), respectively, but these differences were not statistically significant. When comparing the outcome pH between both groups, we also did not find any statistically significant differences between the two interventions (Figure 5). We should interpret these results with caution because only eight patients presented with a diagnosis of hypothyroidism.

## DISCUSSION

To the best of our knowledge, we are not aware of any other randomized trial that analyzed the RGV and its pH by direct aspiration using a gastroscope; and compare two different timings of mannitol ingestion before a colonoscopy, making the study the first of its kind.

The results included non-statistically significant differences for measurements of both RGV and pH, between bowel preparations at 3 and 6 hours before the colonoscopy procedure. This finding is consonant with that obtained among the subgroups of individuals presenting with high and low BMI, Boston scale score values, and patients within the different categories of ASA scores. Patients with a hernia and enlarged hiatus did not show differences in the RGV between the two groups. However, the hypothyroidism subgroup showed a higher RGV, but was not statistically significant. These results differ from those for the subgroup with diabetes mellitus, which presented a higher RGV with statistical significance. We report implications from these findings below.

The analysis reports no significant differences in the levels of RGV and pH for bowel preparation with mannitol administered at 3 and 6 hours before the colonoscopy procedure (Figure 3). Previous studies using laxative agents other than mannitol, support this finding. One of these studies evaluated the RGV and gastric pH in patients receiving day-before bowel preparation compared to those receiving a laxative on the day of the colonoscopy under deep sedation, and concluded that subjects in both groups presented similar values for gastric pH and RGV (21). In another study using PEG bowel preparation, the authors recorded comparable values for the residual volume for both groups of subjects who fasted overnight and those who underwent the procedure 3 hours after the ingestion of the bowel preparation (22,23). This study goes one step further to suggest that undergoing colonoscopy at 3 hours or ≥6 hours after the last ingestion of bowel preparation presented similar results. Gastroenterologists’ day-to-day experience, past studies (24), and the recommendations of the ASA corroborate these findings. The latter states that patients can take clear liquids, including bowel preparations, up to 2 hours before sedation (15). Providers should probably be discouraged from waiting longer than 2 hours after the last mannitol ingestion. The reason for this recommendation is that undergoing colonoscopy between 2 to 3 hours *versus* 8 hours or longer after the last intake of bowel preparation presented with equal advantages with no risk of aspiration from gastric content. A systematic review of practice guidelines on preoperative fluid management and fasting, reported that recommendations to minimize preoperative fasting and ingestion of clear fluids until 2 hours before the administration of anesthesia presented a robust evidence base and strong support for immediate implementation (24). In alignment with these recommendations, we suggest an adherence to shorter fasting periods especially before the colonoscopy procedure, since prolonged fasting protocols may result in adverse events like dehydration and electrolyte imbalance, thus putting the patient at risk.

Based on the subgroup analyses, lean and obese subjects reported similar RGV and pH values. Our finding aligns with previous studies demonstrating identical RGV and pH levels in fasting obese and lean individuals (25). On the contrary, previous studies observed that lean subjects present a smaller volume of acid content in stomach and a higher incidence of combined high-volume and low-pH stomach content compared to their obese counterparts (26). Other articles report a more frequent occurrence of delayed gastric emptying in obese subjects (27-29). Compared to non-obese, morbidly obese subjects reported significantly higher RGV after 8-11 hours of fasting (28,30).

The results indicate that while patients with diabetes mellitus present a significantly higher RGV in the 3-hour group than in the 6-hour group, there were no differences observed with pH levels. There is not much controversy regarding whether diabetes mellitus patients might present with frequent involvement of digestive system, such as gastrointestinal symptoms and abnormal gastrointestinal motility (31). Gastroparesis contributes to the malabsorption of orally-administered medications, ultimately leading to inadequate glycemic control. Some studies oppose the findings, reporting no association between RGV and conditions such as diabetes and gastroparesis (23). However, most of them have reported an association between diabetes mellitus and gastroparesis (31,32), suggesting that autonomic neuropathy (commonly associated with diabetics) and abnormal blood glucose control contributes to the pathogenesis of disordered gastric motility. Regarding the effect of diabetes mellitus on the efficiency of bowel preparation, while the findings suggest that subjects in the 3-hour arm presented a prolonged gastroparesis period compared to patients in the 6-hour arm, many other studies refute this. We can attribute this discrepancy in results to the small sample size of this subgroup in the study.

Like in diabetes mellitus, subjects in the 3-hour intervention arm with hypothyroidism presented a higher RGV. However, there was no statistically significant difference observed between intervention arms. Previous studies that report a significant decrease in gastric activity in hypothyroid patients (33) support this finding. However, studies accessing gastric secretion and emptying in hypothyroidism reported no significant differences in gastric emptying between short-term athyreotic patients and their healthy controls (34).

Although the results of the study are novel, the study has a few limitations. First, as the study population was broad, we did not focus on specific subgroups where we could have identified a more significant difference between the interventions. Elderly patients are one such group, and therefore they should be further studied in future trials. Second, since we excluded some patients, the final balance between randomization arms left two imbalances that could have compromised the validity of the data. In response to this issue, however, we have used adjusted regression models to address the potential imbalance. Finally, as we have mentioned above, the subgroup analyses rely on small sample sizes. Thus our results should be considered preliminary and further validated with more extensive cohort studies focusing on these specific populations.

Despite all the limitations in the subgroup analysis of patients with diabetes and hypothyroidism, we believe that a slightly extended period of fasting should be adopted. We make this recommendation based on the results indicating that the RGV in these patients was higher than in cases where these diseases were not present, even though there was statistical significance only in the subgroup of patients with diabetes.

## CONCLUSIONS

There was no difference between RGV and pH values for bowel preparation with mannitol administration at 3 *versus* 6 hours before endoscopic examinations, except RGV in diabetic patients. As prolonged fasting protocols may result in adverse events such as dehydration and electrolyte imbalance, we can state from the present findings that colonic preparation with mannitol in shorter fasting periods, such as 3 hours, can be adopted safely and routinely.

## AUTHOR CONTRIBUTIONS

Ruiz RF contributed to the conception and design of the study, the analysis and interpretation of the data, and drafting and preparation of the manuscript. Jukemura J, Alves PRA and Santos ML contributed to the conception and design of the study. All authors contributed to revising the manuscript and have read and approved the final version of the manuscript.

## Figures and Tables

**Table 1:** Total study sample stratified by intervention and randomization effectiveness.

Variable	Total (100)	3-hour arm (50)	6-hour arm (50)	Variable
Age (in years) (Mean±SD+)	55.7 (±19.9)	59.7 (±18.7)	51.7 (±20.5)	0.041
Female (%)	51 (51%)	26 (52%)	25 (50%)	0.847
Male (%)	49 (49%)	24 (48%)	25 (50%)	0.843
BMI# (kg/m^2^) (Mean±SD)	27.5 (±5.56)	26.4 (±5.78)	28.5 (±5.15)	0.046
ASA * *score*				1
I (%)	38 (38%)	18 (36%)	16 (32%)	
II (%)	62 (62%)	32 (64%)	34 (68%)	
Boston Scale (Mean±SD)	8.55 (±0.79)	8.51 (±0.86)	8.59 (±0.73)	0.619
6 (%)	4 (3.92%)	1 (1.96%)	3 (5.88%)	
7 (%)	7 (6.86%)	4 (7.84%)	3 (5.88%)	
8 (%)	20 (19.6%)	10 (19.6%)	10 (9.6%)	
9 (%)	71 (69.6%)	36 (70.6%)	35 (68.6%)	
Gastric pH (Mean±SD)	2.15 (±0.58)	2.13 (±0,63)	2.18 (±0.52)	0.676
Logarithm of residual gastric volume (mL) (Mean±SD)	3.72 (±0.88)	3.72 (±0.94)	3.73 (±0.83)	0.96
Residual gastric volume (mL) (Mean±SD)	57.48 (+45.65)	58.69 (+45.48)	56.2 (+46.24)	0.791
Residual gastric volume > 25 mL (%)	75 (73.5%)	39 (76.5%)	36 (70.6%)	0.654
Hernia (%)	12 (12%)	9 (18%)	3 (6%)	1
Enlarged hernia (%)	14 (14%)	8 (16%)	6 (12%)	0.774

+ SD-Standard deviation;

# BMIBody mass index;

* ASA score-American Society of Anthesiologists score.

**Table 2:** Adjusted predicted means and 95% confidence intervals for outcome measures.

Variable	3-hour arm	6-hour arm	*p*
Residual gastric volume (mL)	57.6 (45.9-69.3)	57.4 (45.6-69.1)	0.98
Gastric pH	2.13 (1.97-2.3)	2.17 (2.01-2.34)	0.732
Logarithm of residual gastric volume (mL)	41.9 (33.4-52.6)	40.9 (32.6-51.3)	0.882

The variables are described as mean with 95% confidence intervals given in brackets.

**Table 3:** Effects of intervention on outcomes among eight subgroups of patients.

Variable	3-hour arm	6-hour arm	*p*
BMI#≤26.4 kg/m^2^			
Residual gastric volume (mL)	47.5 (29.8-65.1)	56.5 (41.8-71.2)	*p*=0.455
Logarithm of residual gastric volume (mL)	34.1 (22.6-51.6)	35.7 (25.4-50.4)	*p*=0.868
Gastric pH	2.17 (1.89-2.45)	2.23 (2-2.46)	*p*=0.747
BMI# > 26.4 kg/m^2^			
Residual gastric volume (mL)	69.3 (53.1-85.5)	52.1 (32.7-71.4)	*p*=0.189
Logarithm of residual gastric volume (mL)	53.7 (41.6-69.3)	42.7 (31.4-57.9)	*p*=0.268
Gastric pH	2.17 (1.98-2.36)	2 (1.77-2.23)	*p*=0.262
Boston Scale < 9			
Residual gastric volume (mL)	68.3 (46.9-89.8)	63 (42.2-83.8)	*p*=0.73
Logarithm of residual gastric volume (mL)	56.1 (39.2-80.1)	47.2 (33.4-66.7)	*p*=0.508
Gastric pH	2.04 (1.73-2.36)	2.1 (1.8-2.4)	*p*=0.796
Boston Scale≥9			
Residual gastric volume (mL)	53.2 (38.8-67.5)	54.7 (40.1-69.3)	*p*=0.886
Logarithm of residual gastric volume (mL)	36.5 (27.2-48.9)	38.9 (28.9-52.4)	*p*=0.771
Gastric pH	2.22 (2.02-2.41)	2.16 (1.96-2.35)	*p*=0.688
ASA*-Healthy			
Residual gastric volume (mL)	33.2 (19.6-46.9)	48.1 (34.7-61.4)	*p*=0.147
Logarithm of residual gastric volume (mL)	25.4 (18.5-35)	35.5 (26-48.6)	*p*=0.16
Gastric pH	2.08 (1.82-2.35)	2.23 (1.97-2.49)	*p*=0.459
ASA*-Mild systemic disease			
Residual gastric volume (mL)	74.3 (57-91.7)	62.8 (45.2-80.5)	*p*=0.378
Logarithm of residual gastric volume (mL)	59.4 (43.6-80.9)	43.9 (32-60.1)	*p*=0.194
Gastric pH	2.22 (2.01-2.43)	2.08 (1.86-2.29)	*p*=0.353
Diabetic patients			
Residual gastric volume (mL)	127 (104-149)	43.4 (21-65.9)	*p*<0.001
Logarithm of residual gastric volume (mL)	116 (87.1-155)	37.8 (28.3-50.6)	*p*<0.001
Gastric pH	2.43 (2.1-2.75)	2.15 (1.82-2.48)	*p*=0.26
Hypothyroid patients			
Residual gastric volume (mL)	111 (52.7-170)	63.2 (-19.8-146)	*p*=0.47
Logarithm of residual gastric volume (mL)	139 (47.3-409)	21.7 (4.69-100)	*p*=0.169
Gastric pH	2.33 (1.74-2.92)	2.24 (1.4-3.07)	*p*=0.882

# BMI - Body mass index; *ASA-American Society of Anesthesiologists.

All variables are described as mean with 95% confidence intervals given in brackets.
